# title

**DOI:** 10.1093/geroni/igaa057.1962

**Published:** 2020-12-16

**Authors:** Elliane Irani

**Affiliations:** Case Western Reserve University, Ohio, United States

## Abstract

abstract

